# NADH autofluorescence, a new metabolic biomarker for cancer stem cells: Identification of Vitamin C and CAPE as natural products targeting “stemness”

**DOI:** 10.18632/oncotarget.15400

**Published:** 2017-02-16

**Authors:** Gloria Bonuccelli, Ernestina Marianna De Francesco, Rianne de Boer, Herbert B. Tanowitz, Michael P. Lisanti

**Affiliations:** ^1^ The Paterson Building, University of Manchester, Withington, M20 4BX, United Kingdom; ^2^ Department of Pharmacy, Health and Nutritional Sciences, University of Calabria, Rende, Italy; ^3^ Departments of Pathology and Medicine, Albert Einstein College of Medicine, Bronx, NY, USA; ^4^ Translational Medicine, School of Environment and Life Sciences, Biomedical Research Centre, University of Salford, Greater Manchester, M5 4WT, United Kingdom

**Keywords:** cancer stem-like cells, metabolic heterogeneity, metabolic cell fractionation, mitochondria, NADH

## Abstract

Here, we assembled a broad molecular “tool-kit” to interrogate the role of metabolic heterogeneity in the propagation of cancer stem-like cells (CSCs). First, we subjected MCF7 cells to “metabolic fractionation” by flow cytometry, using fluorescent mitochondrial probes to detect PCG1α activity, as well ROS and hydrogen-peroxide (H2O2) production; NADH levels were also monitored by auto-fluorescence. Then, the various cell populations were functionally assessed for “stem cell activity”, using the mammosphere assay (3D-spheroids). Our results indicate that a sub-population of MCF7 cells, with increased PGC1α activity, high mitochondrial ROS/H2O2 production and high NADH levels, all form mammospheres with a higher efficiency. Thus, it appears that mitochondrial oxidative stress and the anti-oxidant response both contribute to the promotion of mitochondrial biogenesis and oxidative metabolism in CSCs. Further validation was provided by using specific inhibitors to target metabolic processes (the NAD+ salvage pathway, glycolysis, mitochondrial protein synthesis and OXPHOS), significantly reducing CSC propagation. As a consequence, we have now identified a variety of clinically-approved drugs (stiripentol), natural products (caffeic acid phenyl ester (CAPE), ascorbic acid, silibinin) and experimental pharmaceuticals (actinonin, FK866, 2-DG), that can be used to effectively inhibit CSC activity. We discuss the use of CAPE (derived from honey-bee propolis) and Vitamin C, as potential natural therapeutic modalities. In this context, Vitamin C was ∼10 times more potent than 2-DG for the targeting of CSCs. Similarly, stiripentol was between 50 to 100 times more potent than 2-DG.

## INTRODUCTION

Cancer stem-like cells (CSCs) are thought to be the root cause of chemotherapy-resistance and radio-resistance, ultimately leading to treatment failure in patients with advanced disease [1–3]. They have been directly implicated mechanistically in tumor recurrence and metastasis, resulting in poor patient survival [4–6]. However, the metabolic basis of the CSC phenotype remains largely unexplored.

Recently, based on unbiased label-free proteomics analysis, we proposed that mitochondrial biogenesis may be a key driver of the CSC phenotype [7]. A prediction of this hypothesis is that high mitochondrial mass would be a metabolic biomarker for CSCs. To test this hypothesis experimentally, we used a vital dye, called MitoTracker, to stain mitochondria in living cancer cells. This allowed us to purify the Mito-high and the Mito-low cell populations by flow cytometry. Interestingly, the Mito-high cell population showed a clear enrichment of cells with the characteristics of CSCs [8–13]. Thus, the use of “metabolic fractionation”, using mitochondrial-based probes and flow cytometry, is a new successful strategy that also provides a functional assay for dissecting how metabolic heterogeneity contributes to the CSC phenotype.

Here, we have expanded this metabolic fractionation approach to the use of i) other mitochondrial probes (for monitoring mitochondrial biogenesis, ROS production and hydrogen peroxide) and ii) NADH auto-fluorescence. Our results indicate that increased mitochondrial oxidative stress and high NADH levels are both key characteristics of the CSC metabolic phenotype.

This strategic approach also allowed us to identify several new weak points, such as NAD+ depletion, for metabolically targeting CSCs.

## RESULTS

### NAD(P)H auto-fluorescence as a new metabolic biomarker for CSCs

Here, we set out to assess the role of cell metabolism in generating molecular heterogeneity or diversity in cancer cells, with a specific focus on the CSC phenotype.

To address this issue experimentally, we chose to subject the MCF7 cell line to metabolic fractionation via flow cytometry (FACS). For this purpose, we employed a number of fluorescent probes to specifically monitor mitochondrial biogenesis (PGC1α-eGFP), mitochondrial ROS production (Dihydro-Rhodamine 123) and mitochondrial hydrogen-peroxide levels (H2O2; Mito-PY1). In addition, the levels of NAD(P)H were monitored by auto-fluorescence, at an emission wavelength of 470-nm. The experimental utility of these probes is summarized in Table 1.

**Table 1:** Molecular “Tool-Kit” for Investigating the Role of Mitochondria in Metabolic Heterogeneity & Cancer Stem Cells

Process Interrogated	Probe or Inhibitor	Output/Assay
		
Mitochondrial Biogenesis	PGC1ɑ-eGFP	Metabolic Fractionation: Flow Cytometry and 3D-Spheroids
Mitochondrial ROS	Dihydro-Rhodamine 123	Metabolic Fractionation: Flow Cytometry and 3D-Spheroids
Mitochondrial H2O2	Mito-PY1	Metabolic Fractionation: Flow Cytometry and 3D-Spheroids
NADH/NADPH Levels	Auto-fluorescence (470-nm)	Metabolic Fractionation: Flow Cytometry and 3D-Spheroids
		
Mitochondrial Protein Synthesis	Actinonin	3D-Spheroids
NAD(+) Salvage Pathway	FK866	3D-Spheroids
Mitochondrial OXPHOS	CAPE	Seahorse XFe96 Analyzer and 3D-Spheroids
Glycolysis	2-DG, Ascorbic Acid,	3D-Spheroids Silibinin, Stiripentol

**Figure 1:**
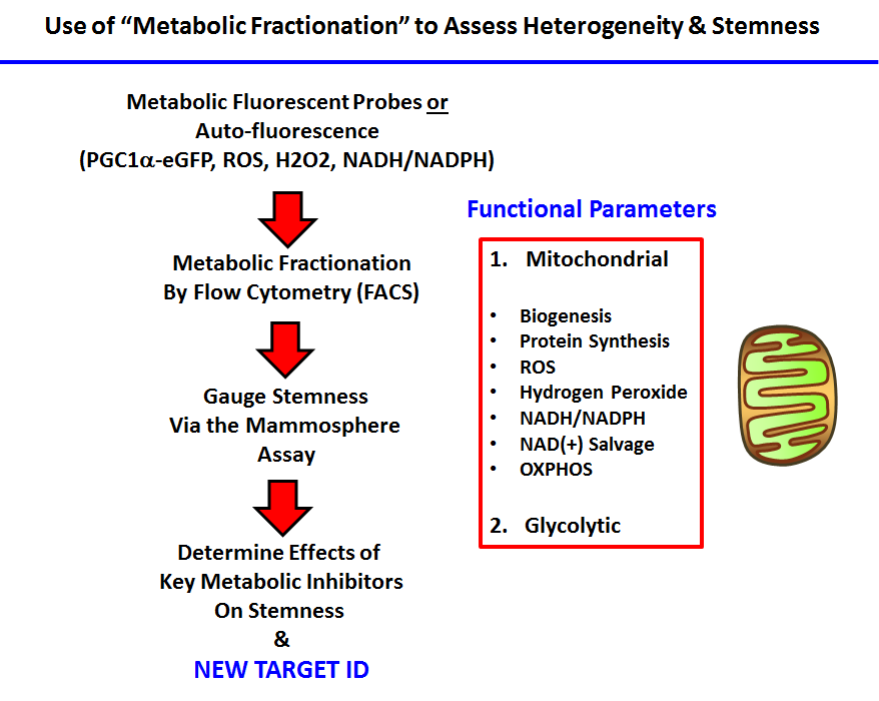
Experimental strategy for examining the role of metabolic heterogeneity in CSC propagation Our overall strategy for investigating the role of mitochondrial metabolism in CSCs, using specific fluorescent probes and metabolic fractionation by flow cytometry (FACS) is outlined. Specific metabolic inhibitors were also employed to directly validate our observations; see Table 1.

In this fractionation scheme, cells containing low- or high-levels of a given vital probe were then analyzed phenotypically, using the mammosphere assay as a measure of “cancer stem cell activity”. A summary of this systematic approach is briefly presented schematically in Figure 1.

**Figure 2:**
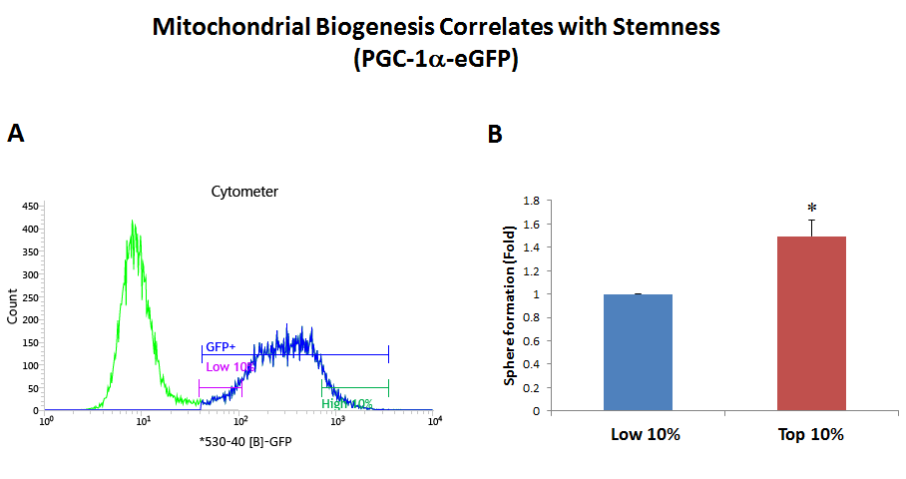
Mitochondrial biogenesis correlates with stemness PGC-1α is a key regulator of energy metabolism, since it stimulates mitochondrial biogenesis. Thus, we generated a stable MCF7 cell line transfected with the promoter of the murine PGC-1α gene linked to eGFP, as a surrogate marker of mitochondrial biogenesis. Therefore, using this reporter system, increased GFP fluorescence is a predictor of increased PGC-1α levels and/or activity. MCF7-PGC-1α-eGFP cells were first subjected to metabolic fractionation via flow cytometry (FACS) to isolate the GFP-high and GFP-low cell populations. These two cell populations were then immediately plated in low-attachment cell culture plates, to assess their capacity for mammosphere formation. Importantly, GFP-high cells showed an ∼1.5-fold increase in their capacity for mammosphere formation, relative to GFP-low cells. These results suggest that there is a clear correlation between increased PGC-1α activity and stemness in cancer cells. GFP-high = highest or top 10% GFP intensity; GFP-low = lowest or bottom 10% GFP intensity. Bar graphs show the mean ± SEM, *t*-test, two-tailed test. *p < 0.05. *N* = 3 independent experiments. A representative FACS tracing is presented in panel **A**. and quantitation of mammosphere formation is shown in panel **B**.

**Figure 3:**
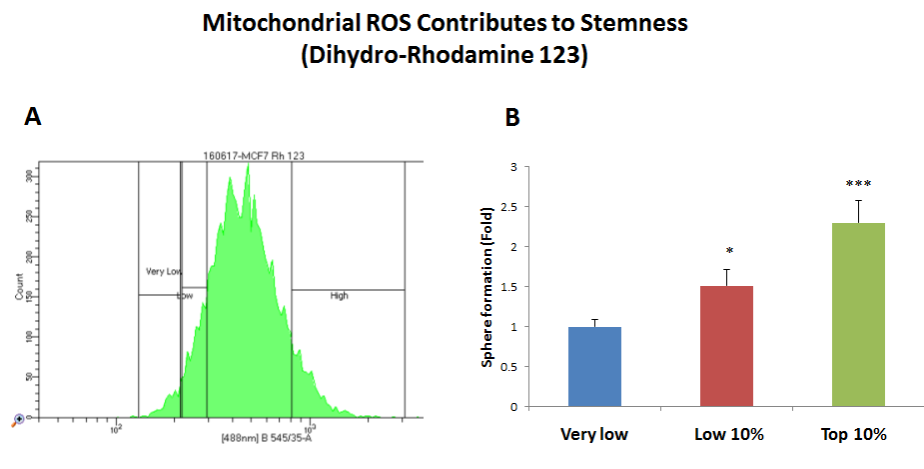
Increased mitochondrial ROS production contributes to stemness MCF7 cells were first live-stained with a fluorescent probe [dihydro-rhodamine 123 (DHR)] to monitor mitochondrial ROS production. Then, DHR-stained MCF7 cells were subjected to metabolic fractionation by flow cytometry (FACS), to isolate the DHR-high, -low and -negative cell populations. Each cell population was then plated in low-attachment cell culture plates, to assess their capacity for mammosphere formation. Note that the DHR-high cell population showed a 2.3-fold increase in mammosphere formation, relative to DHR-negative cells. Therefore, high mitochondrial ROS production directly correlates with CSC activity. DHR-high = highest or top 10% intensity; DHR-low = lowest or bottom 10% intensity; DHR-negative = lowest possible intensity (0 to 4%). Bar graphs show the mean ± SEM, *t*-test, two-tailed test. *p < 0.05, ***p < 0.0001. *N* = 3 independent experiments. A representative FACS tracing is presented in panel **A**. and quantitation of mammosphere formation is shown in panel **B**.

**Figure 4:**
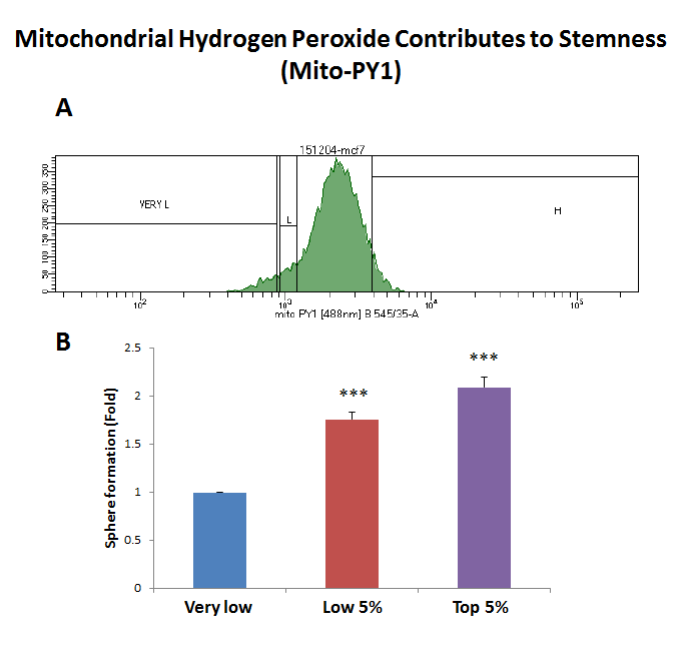
Increased mitochondrial hydrogen peroxide (H2O2) production contributes to stemness MCF7 cells were first live-stained with a fluorescent probe [Mitochondria peroxy yellow 1 (MPY1)] to monitor mitochondrial H2O2 production. Then, MPY1-stained MCF7 cells were subjected to metabolic fractionation by flow cytometry (FACS), to isolate the MPY1-high, -low and -negative cell populations. Each cell population was then plated in low-attachment cell culture plates, to assess their capacity for mammosphere formation. Note that the MPY1-high cell population showed a 2.1-fold increase in mammosphere formation, relative to MPY1-negative cells. Therefore, high mitochondrial H2O2 production directly correlates with CSC activity. MPY1-high = highest or top 5% intensity; MPY1-low = lowest or bottom 5% intensity; MPY1-negative = lowest possible intensity (0 to 2%). Bar graph shows the mean ± SEM, *t*-test, two-tailed test. ***p < 0.0001. *N* = 3 independent experiments. A representative FACS tracing is presented in panel **A**. and quantitation of mammosphere formation is shown in panel **B**.

**Figure 5:**
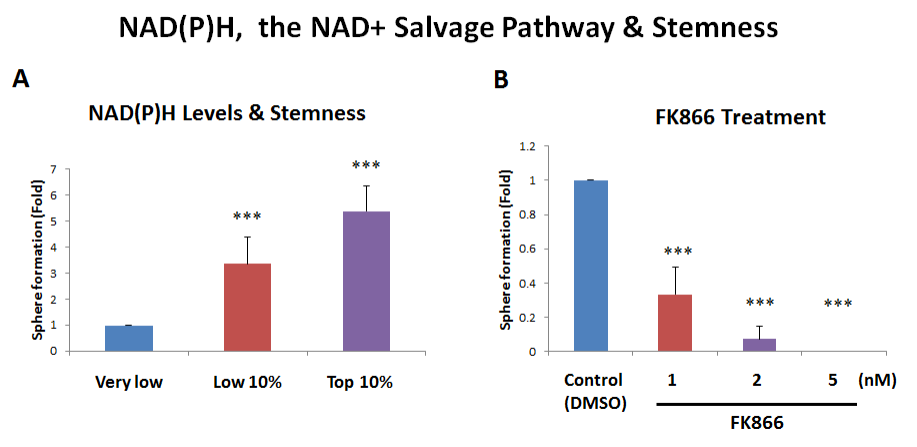
Increased NAD(P)H levels directly correlate with stemness **A**. NAD(P)H auto-fluorescence. NAD(P)H levels are known to directly correlate with mitochondrial oxidative capacity (OXPHOS), as well as anti-oxidant capacity. NAD(P)H generates a significant amount of cellular auto-fluorescence and is detectable by its emission peak at 470-nm. To examine the possible contribution of NAD(P)H to stemness, MCF7 cells were subjected to metabolic fractionation by flow cytometry (FACS), to isolate the NAD(P)H-high, -low and -negative cell populations. Each cell population was then plated in low-attachment cell culture plates, to assess their capacity for mammosphere formation. Note that the NAD(P)H-high cell population showed a 5.4-fold increase in mammosphere formation, relative to the NAD(P)H-negative cells. Therefore, high cellular NAD(P)H levels directly correlate with CSC activity. NAD(P)H-high = highest top 10% intensity; NAD(P)H-low = lowest bottom 10% intensity; NAD(P)H-negative = lowest possible intensity (no auto-fluorescence). **B**. Effects of the inhibitor FK866. To induce cellular depletion of NAD(P)H, we used a known inhibitor of the NAD+ salvage pathway. Note that treatment with FK866 dose-dependently inhibited mammosphere formation, with an IC-50 of less than 1 nM. Bar graphs are shown as the mean ± SEM, *t*-test, two-tailed test. ***p < 0.0001. *N* = 3 independent experiments.

Interestingly, using this reductionistic experimental approach, our results directly show that a particular sub-population of MCF7 cells, with the highest levels of mitochondrial biogenesis, mitochondrial ROS/H2O2, and NADH levels, have the highest capacity to efficiently generate mammospheres (Figures 2–5).

As NAD(P)H auto-fluorescence is an intrinsic property of cells and does not require the use of any exogenous fluorescent probes, we propose that NAD(P)H auto-fluorescence could be used as a new metabolic biomarker for CSC enrichment (Figure 5A). Importantly, high levels of NAD(P)H auto-fluorescence are known to be a surrogate marker for mitochondrial “power”, high OXPHOS capacity and increased ATP production. As such, CSCs may be strictly dependent on NAD(P)H to maintain their enhanced mitochondrial function.

As further functional validation, we next examined the sensitivity of MCF7 mammosphere formation to NAD+ depletion, by employing the well-characterized NAMPT inhibitor, namely FK866 [14]. Figure 5B shows that mammosphere formation was dramatically inhibited by FK866, with an IC-50 of less than 1 nM. Thus, an intact NAD+ salvage pathway is strictly required for mammosphere formation, supporting our results using NAD(P)H auto-fluorescence, which enriched CSC activity by more than 5-fold.

**Figure 6:**
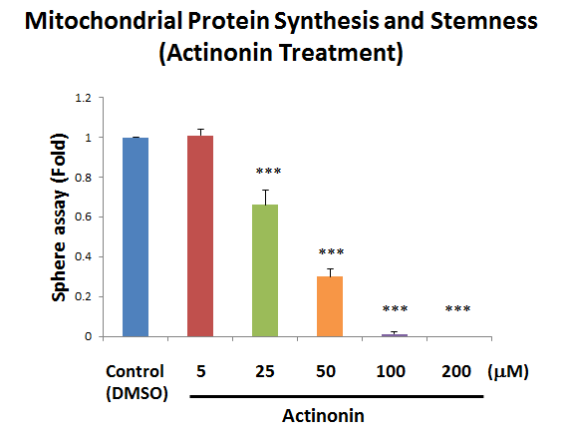
Actinonin, a naturally-occurring inhibitor of mitochondrial protein synthesis, blocks mammosphere formation Actinonin is a naturally-occurring antibiotic. However, actinonin also functions as an inhibitor of mitochondrial protein translation, by targeting a human mitochondrial protein, known as peptide deformylase (abbreviated as PDF). PDF is required to remove the formyl-group from the initiator methionine of mitochondrial proteins synthesized on mitochondrial ribosomes. Note that actinonin inhibited mammosphere formation, with an IC-50 between 25 and 50 μM. The bar graph shows the mean ± SEM, *t*-test, two-tailed test. ***p < 0.0001. *N* = 3 independent experiments.

Similarly, as further validation, we also show that a known selective inhibitor of mitochondrial protein synthesis (the natural antibiotic actinonin; [15]), efficiently blocked mammosphere formation, with an IC-50 between 25 and 50 μM (Figure 6). This is consistent with our observations regarding a significant increase in mitochondrial biogenesis, as reflected by our studies using the PGC1α-eGFP probe.

### Identification of vitamin C as a natural product targeting “stemness”

Since the NAMPT inhibitor FK866 suffers from a number of serious side effects, including thrombocytopenia, lymphopenia and anemia, as well as severe nausea and mild fatigue [16], we sought to identify other potentially less toxic metabolic approaches for targeting CSCs.

For this purpose, we examined the activity of a number of clinically-approved drugs, natural products, as well as experimental pharmaceuticals. Since glycolysis is especially critical for maintaining the TCA cycle, OXPHOS and overall mitochondrial function, we next assessed the effects of known glycolytic inhibitors.

**Figure 7:**
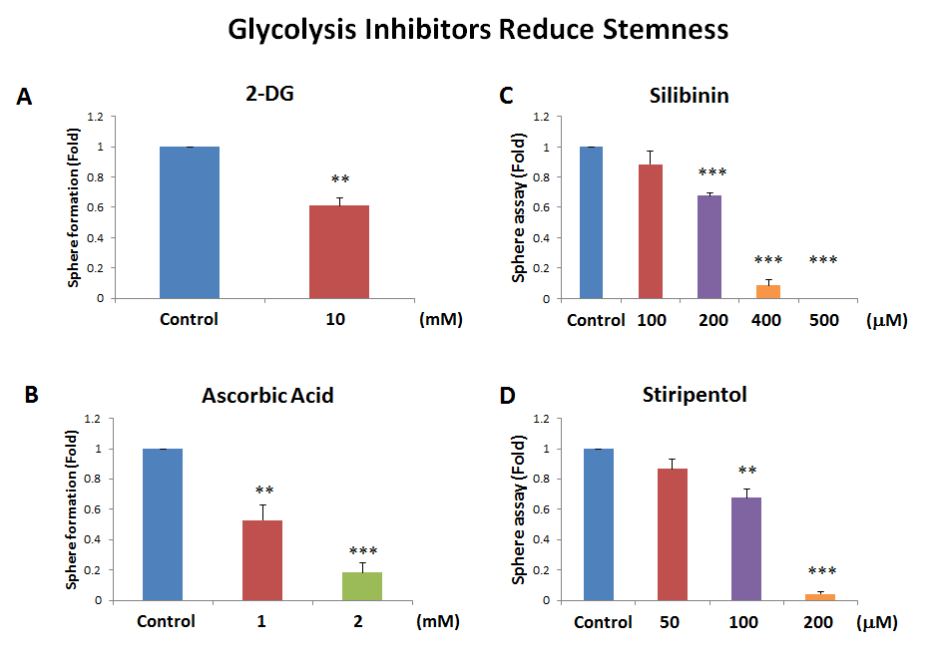
Glycolysis inhibitors also block mammosphere formation Here, we examined if 4 distinct glycolysis inhibitors have the capacity to block mammosphere formation: **A**. 2-deoxy-glucose (2-DG); **B**. vitamin C (ascorbic acid); **C**. silibinin; and **D**. stiripentol. Note that the two most potent glycolysis inhibitors that we identified were silibinin and stiripentol. Silibinin, which functionally blocks glucose uptake, inhibited mammosphere formation, with an IC-50 between 200 and 400 μM. Similarly, the FDA-approved drug stiripentol, which behaves as an LDHA inhibitor, blocked mammosphere formation with an IC-50 between 100 and 200 μM. Bar graphs are shown as the mean ± SEM, *t*-test, two-tailed test. **p < 0.005, ***p < 0.0001. *N* = 3 independent experiments.

Interestingly, our results directly show that the classical glycolysis inhibitor, namely 2-deoxy-glucose (2-DG), inhibits mammosphere formation, with an IC-50 near 10 mM (Figure 7A). Similarly, we show that two other natural products that function as effective glycolysis inhibitors, also inhibited mammosphere formation. More specifically, vitamin C (ascorbic acid), which induces oxidative stress and inhibits the activity of GAPDH (a key glycolytic enzyme) [17], also inhibited mammosphere formation, with an IC-50 of 1 mM (Figure 7B). Therefore, vitamin C was ∼10 times more potent than 2-DG at targeting CSC propagation. Interestingly, silibinin (the major active constituent of silymarin, an extract of milk thistle seeds) [18], which specifically functions as an inhibitor of glucose uptake, blocked mammosphere formation, with an IC-50 between 200 and 400 μM (Figure 7C). Finally, the FDA-approved drug stiripentol, which behaves as an LDHA inhibitor [19], blocked mammosphere formation with an IC-50 between 100 and 200 μM (Figure 7D). Thus, stiripentol was the most potent glycolysis inhibitor that we identified for targeting CSCs and it was 50 to 100 times more potent than 2-DG.

### Identification of CAPE as a natural product that metabolically targets mitochondria and CSC activity

Honey-bee propolis (a.k.a., “bee glue”) is a resinous-like substance that bees manufacture as a sealant for filling unwanted crevices in their hive [20]. Propolis has a strong history of medicinal use, dating back more than 2,000 years [20–22]. In fact, caffeic acid phenyl ester (CAPE), a key component of honey-bee propolis, has potent anti-cancer properties [22]. However, CAPE's exact mechanism of action remains largely unknown.

**Figure 8:**
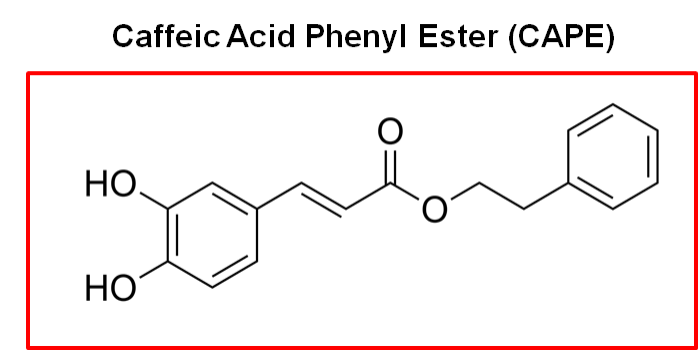
Chemical structure of caffeic acid phenyl ester (CAPE), a key component of honey-bee propolis The presence of two aromatic rings in CAPE conveys significant hydrophobicity upon the molecule, which could increase its affinity for cellular membranes, such the mitochondrial membrane.

**Figure 9:**
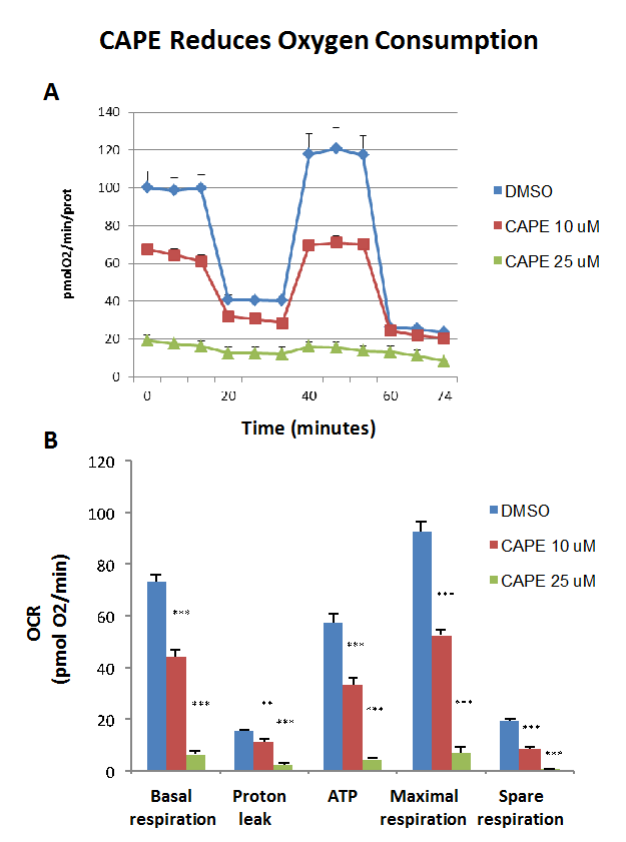
Treatment with CAPE lowers mitochondrial respiration The metabolic OCR profile of MCF7 cells grown in monolayers, after 72 hours of treatment with CAPE, was examined using the Seahorse XFe96 analyser (Top panel, representative experiment). The bar graph, in the Lower panel, shows that the all the parameters relative to the oxygen consumption (OCR), are significantly decreased by both concentration of CAPE, 10 μM and 25 μM, as compared to the vehicle-treated control cells (DMSO). The bar graph shows the mean ± SEM, *t*-test, two-tailed test. **p < 0.005, ***p < 0.0001. *N* = 3 independent experiments.

**Figure 10:**
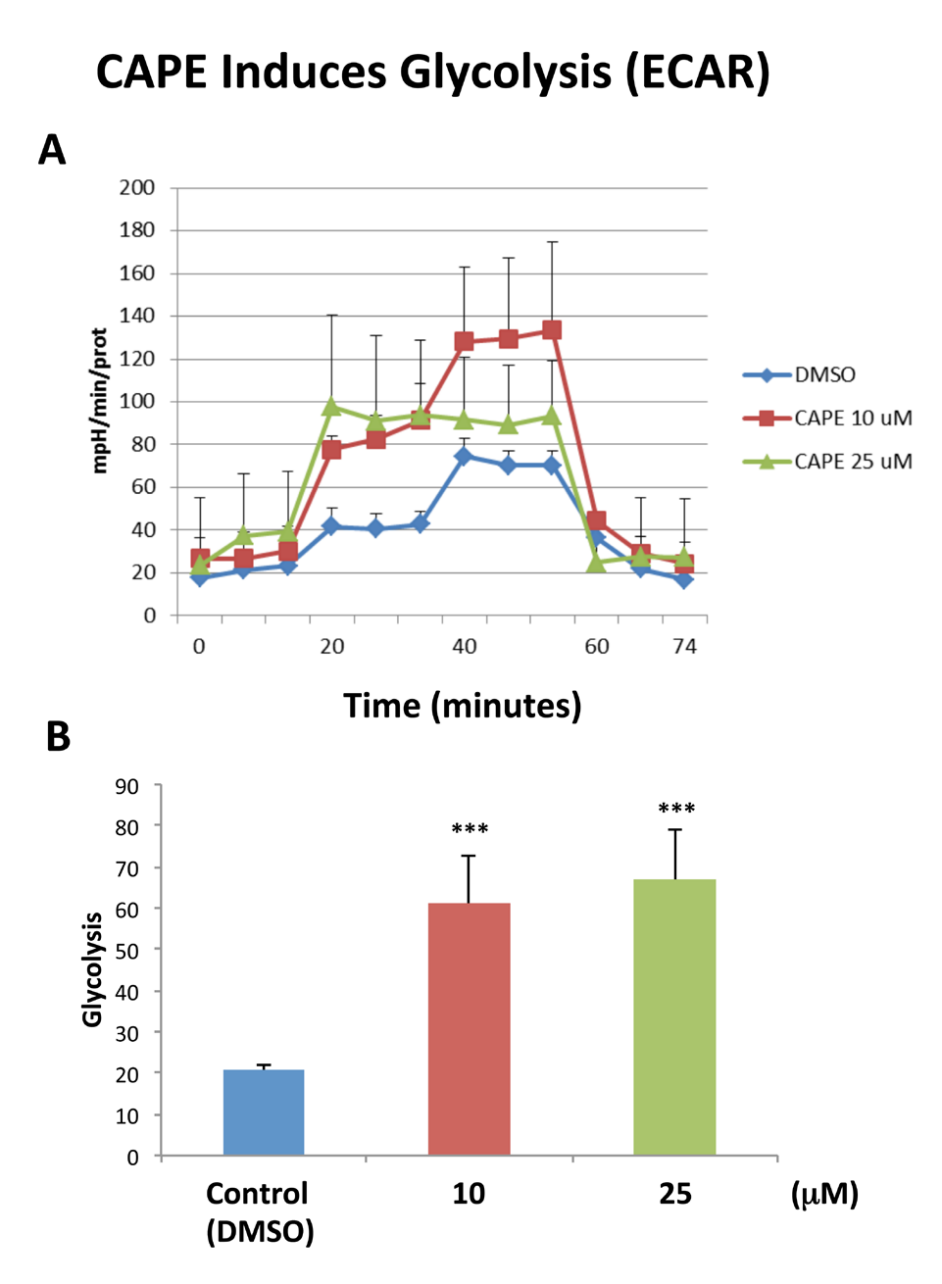
Treatment with CAPE increases glycolysis The metabolic ECAR profile of MCF7 cells grown in monolayers, after 72 hours of treatment with CAPE, was examined using the Seahorse XFe96 analyser (Top panel, combination of three experiments). The bar graph, in the Lower panel, shows that glycolysis (measured after addition of D-glucose) is increased after treatment with CAPE at the concentrations of 10 μM and 25 μM, as compared to the vehicle-treated control cells (DMSO). Data are presented in the graph as combination of *N* = 3 independent experiments. Bar graphs are shown as the mean ± SEM, *t*-test, two-tailed test. ***p < 0.0001. *N* = 3 independent experiments.

Because of it aromatic ring structure (Figure 8), we speculated that CAPE might function as a potent inhibitor of oxidative mitochondrial metabolism. To test this hypothesis, we examined its metabolic effects on MCF7 cell in culture, using the Seahorse XFe96 Metabolic Flux Analyzer. Indeed, Figures 9 and 10 directly show that CAPE quantitatively inhibits the mitochondrial oxygen consumption rate (OCR) and, in turn, induces the onset of aerobic glycolysis (ECAR).

**Figure 11:**
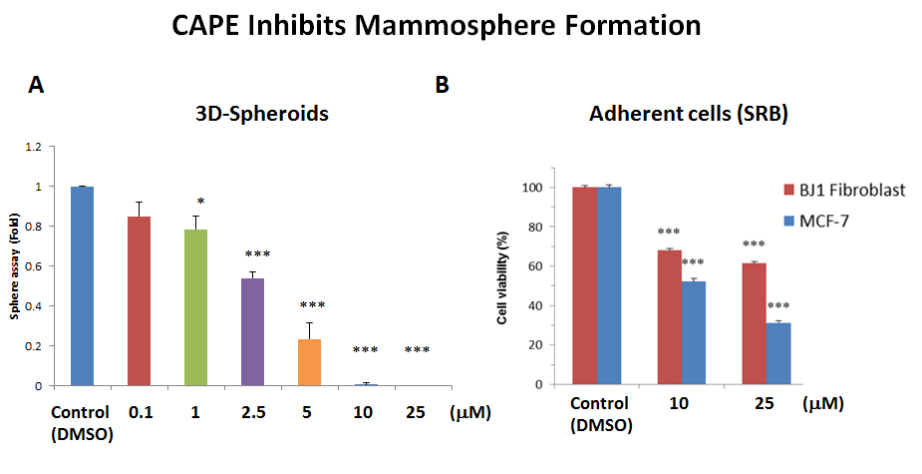
CAPE potently blocks mammosphere formation **A**. 3D-sphere formation. MCF7 cells were plated in low-attachment cell culture plates (6-well format), to assess the ability of CAPE to inhibit mammosphere formation. Note that CAPE treatment was sufficient to significantly reduce mammosphere formation, with an IC-50 of approximately 2.5 μM. **B**. Cell viability. The effects of CAPE on the viability of adherent cells (MCF7 cells and hTERT-BJ1 fibroblasts) was assessed in parallel, using the SRB assay, in a 96-well format. Note that CAPE significantly reduces the viability of MCF7 cells, with an IC-50 of 10 μM. Similar results were obtained with hTERT-BJ1 cells. Cell viability was assessed after 72 hours of treatment. Bar graphs are shown as the mean ± SEM, *t*-test, two-tailed test. *p < 0.05, ***p < 0.0001. *N* = 3 independent experiments.

Next, we tested the effects of CAPE on the ability of MCF7 cells to form 3D mammospheres, under anchorage independent growth conditions. Interestingly, Figure 11A illustrates that CAPE significantly reduces mammosphere formation, with an IC-50 of approximately 2.5 μM. However, it was less effective on adherent “bulk” cancer cells, with an IC-50 of 10 μM (Figure 11B). As such, CAPE showed a 4-fold selectivity for targeting CSC propagation, as compared with adherent MCF7 monolayer cells. In addition, at a concentration of 25 μM, CAPE was twice as toxic for MCF7 cancer cells, as compared with normal fibroblasts. At this concentration, ∼60% of normal fibroblasts survived, while >70% of cancer cells were eliminated. Thus, CAPE shows a clear selectivity for targeting CSCs and adherent cancer cells, relative to normal fibroblasts.

**Figure 12:**
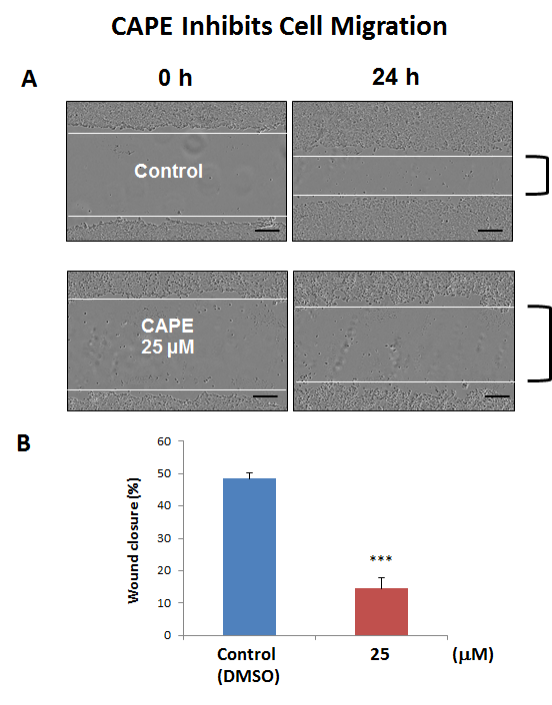
CAPE decreases the migratory capacity of MCF7 cells MCF7 cell migration was monitored using an *in vitro* scratch assay. Images were acquired with the IncuCyte Zoom instrument at 0 and 24 hours. The Upper panel of the figure shows representative images. MCF7 were treated with or without CAPE, at a concentration of 25 uM. Note that CAPE-treated MCF7 cells migrated slower, as compared to vehicle-treated MCF7 cells. In the Lower panel, quantification of cell migration is presented as % wound-closure. Bar graphs are shown as the mean ± SEM, *t*-test, two-tailed test. ***p < 0.0001. Scale bar = 250 μm. *N* = 3 independent experiments.

Often CSCs are associated with an epithelial mesenchymal transition (EMT) phenotype, resulting in an increased capacity for cell migration [23]. As a consequence, we also tested the effects of CAPE on MCF7 cell migration, using a standard “scratch assay”. In this assay system, cell migration is monitored via “wound closure”. Interestingly, Figure 12 shows that treatment with CAPE (25 μM for 24 hours) significantly reduced “wound closure” by ∼70%.

Thus, we conclude that CAPE functions as a “natural” mitochondrial OXPHOS inhibitor, that preferentially targets the CSC sub-population. This could explain CAPE's known anti-cancer properties.

## DISCUSSION

**Figure 13:**
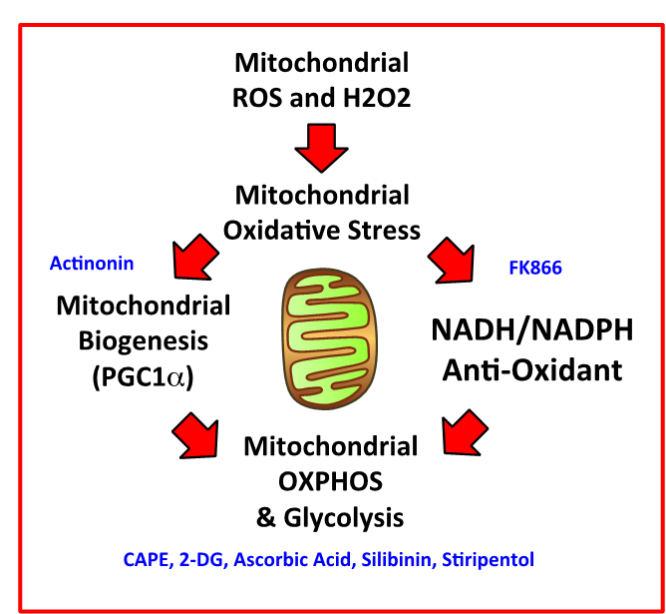
Mitochondrial oxidative stress drives mitochondrial biogenesis and the anti-oxidant response, leading to increased mitochondrial metabolism and stemness We summarize here our overall findings schematically, with a focus on how mitochondrial ROS and H2O2 production may be a trigger, for driving mitochondrial oxidative stress, ultimately leading to increased mitochondrial oxidative metabolism in CSCs. Pharmaceuticals, chemical inhibitors, or natural products that we have identified to target CSCs, which interfere with a specific metabolic process or function, are shown in BLUE.

Here, we interrogated how metabolic heterogeneity plays a key role in the generation of CSCs. More specifically, we employed metabolic fractionation by flow cytometry, to assess the potential role of i) mitochondrial biogenesis, ii) oxidative stress (ROS and H2O2 production) and iii) anti-oxidants, such as NAD(P)H, in CSC propagation (Figure 13). Our data directly shows that a small fraction of the total cell population, characterized by increased PGC1α activity, high mitochondrial ROS/H2O2 and high NADH levels, has the ability to survive and grow under anchorage-independent conditions, driving mammosphere formation. In order to carefully validate our observation, we used a battery of selective metabolic inhibitors to target specific energetic pathways, significantly reducing and/or eliminating CSC propagation. Thus, we have now identified a number of clinically-approved drugs, natural substances and experimental drugs that can be used to effectively inhibit CSC activity. We highlight the utility of certain natural products, such as Silibinin, Vitamin C and CAPE, that could be used to therapeutically target CSCs. Silibinin is the major active component of silymarin, which is an extract prepared from milk thistle seeds.

### NADH auto-fluorescence: A new biomarker for CSCs

NADH fluorescence is the dominant form of auto-fluorescence observed in living cells [24,25]. Interestingly, previous studies have shown that normal intestinal crypt stem cells have the highest levels NADH auto-fluorescence [26], relative to other intestinal cell types. So, high NADH is a property that is conserved between normal and cancerous stem cells. Previous studies have also shown that when non-CSCs and CSCs are both fed mitochondrial fuels (such as L-lactate or ketone bodies), that CSCs quantitatively produce more NADH in response to this stimulus [27]. Similarly, Heeschen and colleagues have previously shown that FAD auto-fluorescence is also an excellent biomarker for CSCs [28]; however, they did not examine the utility of NADH as a biomarker for CSCs in their studies [29]. Interestingly, high levels of NADH auto-fluorescence are a known to be a surrogate marker for mitochondrial “power”, high OXPHOS capacity and increased ATP production. Thus, CSCs may be strictly dependent on NADH to maintain their enhanced mitochondrial function. In accordance with this hypothesis, NAD+ depletion, using the NAMPT inhibitor FK866, potently blocked mammosphere formation, with an IC-50 of less than 1 nM.

### Vitamin C: Targeting metabolism and glycolysis in CSCs

The Noble Prize winner, Linus Pauling, was among the first to describe and clinically test the efficacy of Vitamin C, as a relatively non-toxic anti-cancer agent [30]. More recently, Lew Cantley's group has revisited the mechanism(s) by which Vitamin C targets cancer cells [17]. Interestingly, they showed that Vitamin C has two mechanisms of action. First, it is a potent pro-oxidant, that actively depletes the reduced glutathione pool, leading to cellular oxidative stress and apoptosis in cancer cells. Moreover, it also behaves as an inhibitor of glycolysis, by targeting the activity of GAPDH, a key glycolytic enzyme. However, its effects on CSC activity was not previously evaluated. Here, we show that Vitamin C can also be used to target the CSC population, as it is an inhibitor of energy metabolism that feeds into the mitochondrial TCA cycle and OXPHOS. Similar results were also obtained with 3 other glycolysis inhibitors, namely 2-DG, silibinin and stiripentol. Importantly, stiripentol is a clinically-approved drug, but its use is mainly restricted to the treatment of epileptic seizures in children, and not for cancer therapy [19]. Thus, Vitamin C may prove to be promising agent for new clinical trials, aimed at testing its ability to reduce CSC activity in cancer patients, as an add-on to more conventional therapies, to prevent tumor recurrence, further disease progression and metastasis. Interestingly, a breast cancer based clinical study has already shown that the use of Vitamin C, concurrent with or within 6 months of chemotherapy, significantly reduces both tumor recurrence and patient mortality [31,32]. However, the mechanism underlying its potential clinical benefit remained obscure. Similarly, Vitamin C treatment inhibits tumor growth in murine animal models *in vivo* [33].

### CAPE: Targeting mitochondrial OXPHOS in CSCs

CAPE is another natural product that we identified which mechanistically targets oxidative mitochondrial metabolism and, as a consequence, CSC activity. We directly show, using the Seahorse metabolic flux analyzer, that CAPE quantitatively reduces mitochondrial oxygen consumption (OCR), while inducing a reactive increase in glycolysis (ECAR). As such, it potently inhibits mammosphere formation with an IC-50 of ∼2.5 μM. Similarly, it also significantly inhibits cell migration. Therefore, further metabolic studies using CAPE and other natural components of honey-bee propolis may be warranted.

## CONCLUSIONS

In summary, we have discovered that NAD(P)H auto-fluorescence and several mitochondrial-based fluorescent probes can all be employed to enrich for a population of cells with the characteristics of CSCs. In accordance with these observations, we also demonstrate that 7 different inhibitors of key energetic pathways can be used to effectively block CSC propagation, including three natural products (silibinin, ascorbic acid and CAPE). Future studies will be necessary to test their potential for clinical benefit in cancer patients.

## MATERIALS AND METHODS

### Materials

MCF7 cells were originally purchased from the ATCC. The lentiviral vector encoding the promoter of the murine PGC1α gene, linked to the eGFP reporter, and containing a puromycin-resistance cassette, was custom synthesized by Genecopoeia™ (Maryland, USA; mPGC1α-eGFP-Puro-R). This construct was then used to stably transduce MCF7 cells. Media for cell culture was DMEM (D6546, Sigma-Aldrich). Cell culture media (DMEM/F12) for spheroid culture was purchased from Life Technologies. Caffeic acid phenetyl ester (CAPE), sulforhodamine B (SRB), the glycolytic inhibitor 2-Deoxy-D-Glucose (2-DG), L-ascorbic acid, mitochondria peroxy yellow 1 (Mito-PY1), dihydro-rhodamine 123, and FK866 hydrochloride hydrate, were all purchased from Sigma-Aldrich.

### Generation of stably-transduced MCF7 cells

MCF7 cells were transduced with a lentiviral vector encoding the following reporter plasmid: mPGC1α-eGFP-Puro-R. Briefly, the lentiviral vector was transfected into the 293Ta packaging cells, following the manufacturer's instructions. Two days post-transfection, the cell culture supernatant was collected and centrifuged and used to infect MCF7 cells. After lentiviral transduction, MCF7 cells were selected for 12 days in puromycin (2 μg/ml).

### FACScan analysis and sorting

MCF7 cells were subjected to metabolic fractionation using either the BD FACSAria™ III and BD Influx™ cells sorters. Data were analyzed using FlowJo software (Tree Star, Ashland, OR). Different cell populations were isolated and subjected to mammosphere analysis.

### Mammosphere formation (3D-Spheroid culture)

A single cell suspension was prepared using enzymatic (1x Trypsin-EDTA, Sigma Aldrich, #T3924), and manual disaggregation (25 gauge needle) was used to create a single cell suspension. Five thousand cells were plated with in mammosphere medium (DMEM-F12/B27/20ng/ml EGF/PenStrep), under non-adherent conditions, in six wells plates coated with 2-hydroxyethylmethacrylate (poly-HEMA, Sigma, #P3932) [34]. Cells were grown for 5 days and maintained in a humidified incubator at 37°C at an atmospheric pressure in 5% (v/v) carbon dioxide/air. After five days of culture, the number of spheres with diameter >50 μm were counted.

### Mitochondrial ROS production

To measure mitochondrial ROS production, we used a well-known fluorogenic vital probe, namely dihydro-rhodamine 123 (Cat. No. D1054). For this purpose, samples containing 5 × 10^6^ MCF7 cells (volume 500 μl) were incubated for 10 min at 37°C, together with dihydro-rhodamine 123 at 10 μM. Then, cold PBS was added and the samples were FACS sorted based on the intensity of the fluorochrome. The three cell populations obtained by sorting were seeded under non-adherent conditions and tested for their mammosphere- forming ability.

### Mitochondrial hydrogen peroxide production

For the detection of mitochondrial hydrogen peroxide production, we used Mito-PY1 as a vital fluorescent dye (Cat. No. SML0734). Live cells under adherent conditions were labelled with Mito-PY1 (10 μM) in a PBS solution. After 45 min, Mito-PY1 was removed and replaced with fresh warm PBS. The washes were repeated one more time and the cells were trypsinized, resuspended in PBS and were separated by flow cytometry. Based on their fluorescence intensity, the MCF7 were fractionated and sorted into three distinct cell populations: very low fluorescent cells, low 5% and top 5% GFP cells. The cells were immediately plated under non-adherent conditions at the density of five thousands cells in each well of a six wells-plate to evaluate their mammosphere-forming ability.

### NAD(P)H measurements and treatment with FK866

To optically monitor the levels of mitochondrial OXPHOS in MCF7 cells, it is possible to measure NADH auto-fluorescence by FACS analysis [24,25]. After UV excitation, NADH emits auto-fluorescence at 470-nm. So, we used the fluorescence properties of NADH to sort MCF7 cells into three distinct populations, based on their cellular auto-fluorescence.

Afterwards, we tested their mammosphere-forming ability. Furthermore, we investigated the effect of FK866, a potent inhibitor of the NAD+ salvage pathway biosynthesis, on the mammosphere-forming ability of the sorted cell populations. As it is difficult to distinguish between NADH and NADPH auto-fluorescence (both emit at 470-nm), the values observed represent the sum of these two molecules: NAD(P)H auto-fluorescence. In contrast, FAD emits auto-fluorescence at 525-nm. Thus, NAD(P)H and FAD auto-fluorescence are distinct.

### Metabolic flux analysis

Extracellular acidification rates (ECAR) and oxygen consumption rates (OCR) were analysed using the Seahorse XFe96 bioenergetic analyzer (Seahorse Bioscience, MA, USA). MCF7 cells were maintained in DMEM supplemented with 10% FBS (fetal bovine serum), 2 mM GlutaMAX, and 1% Pen- Strep. Fifteen thousand MCF7 cells were seeded per well, into XFe96-well cell culture plates, and incubated at 37°C in a 5% CO2 humidified atmosphere. After 72 hours, MCF7 cells were washed in pre-warmed XF assay media, as previously described. ECAR and OCR measurements were normalized for cell protein content, by the SRB colorimetric assay. Data sets were analyzed using XFe-96 software and Excel software.

### Cell migration: *in vitro* scratch assay

MCF7 cells were seeded in 12 wells-plates to create a confluent monolayer. Cell monolayers were then scraped to create a scratch with a p-200 pipet tip. Plates were incubated in the IncuCyte™ Zoom (Essen Bioscience) in 5% CO_2_ at 37°C for 24 hours. Complete culture medium containing 10% FBS, -/+ CAPE 25 μM was used. The images were acquired and scanned over a period of 24 hours. Values for migration were obtained after scanning the whole wells (with a 4X objective) and represent the average of three independent experiments. The rate of the migration was evaluated using Image-J software, to measure “wound closure”.

### Statistics

All data are presented as the mean ± SEM. The Student's t-test was used. P < 0.05 was considered statistically significant.

## References

[1] Yu Z, Pestell TG, Lisanti MP, Pestell RG (2012). Cancer stem cells. Int J Biochem Cell Biol.

[2] Brooks MD, Burness ML, Wicha MS (2015). Therapeutic Implications of Cellular Heterogeneity and Plasticity in Breast Cancer. Cell Stem Cell.

[3] Luo M, Clouthier SG, Deol Y, Liu S, Nagrath S, Azizi E, Wicha MS Breast cancer stem cells: current advances and clinical implications. Methods Mol Biol.

[4] Ozsvári B, Lamb R, Lisanti MP (2016). Repurposing of FDA-approved drugs against cancer - focus on metastasis. Aging (Albany: NY).

[5] Martinez-Outschoorn UE, Peiris-Pagés M, Pestell RG, Sotgia F, Lisanti MP (2017). Cancer metabolism: a therapeutic perspective. Nat Rev Clin Oncol.

[6] Peiris-Pagès M, Martinez-Outschoorn UE, Pestell RG, Sotgia F, Lisanti MP (2016). Cancer stem cell metabolism. Breast Cancer Res.

[7] Lamb R, Harrison H, Hulit J, Smith DL, Lisanti MP, Sotgia F (2014). Mitochondria as new therapeutic targets for eradicating cancer stem cells: Quantitative proteomics and functional validation via MCT1/2 inhibition. Oncotarget.

[8] De_Luca A, Fiorillo M, Peiris-Pagès M, Ozsvari B, Smith DL, Sanchez-Alvarez R, Martinez-Outschoorn UE, Cappello AR, Pezzi V, Lisanti MP, Sotgia F (2015). Mitochondrial biogenesis is required for the anchorage-independent survival and propagation of stem-like cancer cells. Oncotarget.

[9] Farnie G, Sotgia F, Lisanti MP (2015). High mitochondrial mass identifies a sub-population of stem-like cancer cells that are chemo-resistant. Oncotarget.

[10] Lamb R, Bonuccelli G, Ozsvári B, Peiris-Pagès M, Fiorillo M, Smith DL, Bevilacqua G, Mazzanti CM, McDonnell LA, Naccarato AG, Chiu M, Wynne L, Martinez-Outschoorn UE, Sotgia F, Lisanti MP (2015). Mitochondrial mass, a new metabolic biomarker for stem-like cancer cells: Understanding WNT/FGF-driven anabolic signaling. Oncotarget.

[11] Fiorillo M, Lamb R, Tanowitz HB, Mutti L, Krstic-Demonacos M, Cappello AR, Martinez-Outschoorn UE, Sotgia F, Lisanti MP (2016). Repurposing atovaquone: targeting mitochondrial complex III and OXPHOS to eradicate cancer stem cells. Oncotarget.

[12] Fiorillo M, Lamb R, Tanowitz HB, Cappello AR, Martinez-Outschoorn UE, Sotgia F, Lisanti MP (2016). Bedaquiline, an FDA-approved antibiotic, inhibits mitochondrial function and potently blocks the proliferative expansion of stem-like cancer cells (CSCs). Aging Albany NY.

[13] Lamb R, Ozsvari B, Lisanti CL, Tanowitz HB, Howell A, Martinez-Outschoorn UE, Sotgia F, Lisanti MP (2015). Antibiotics that target mitochondria effectively eradicate cancer stem cells, across multiple tumor types: Treating cancer like an infectious disease. Oncotarget.

[14] Tolstikov V, Nikolayev A, Dong S, Zhao G, Kuo MS (2014). Metabolomics analysis of metabolic effects of nicotinamide phosphoribosyltransferase (NAMPT) inhibition on human cancer cells. PLoS One.

[15] Lee MD, She Y, Soskis MJ, Borella CP, Gardner JR, Hayes PA, Dy BM, Heaney ML, Philips MR, Bornmann WG, Sirotnak FM, Scheinberg DA (2004). Human mitochondrial peptide deformylase, a new anticancer target of actinonin-based antibiotics. J Clin Invest.

[16] von_Heideman A, Berglund A, Larsson R, Nygren P (2010). Safety and efficacy of NAD depleting cancer drugs: results of a phase I clinical trial of CHS 828 and overview of published data. Cancer Chemother Pharmacol.

[17] Yun J, Mullarky E, C Lu, Bosch KN, Kavalier A, Rivera K, Roper J, Chio II, Giannopoulou EG, Rago C, Muley A, Asara JM, Paik J, Elemento O, Chen Z, Pappin DJ, Dow LE, Papadopoulos N, Gross SS, Cantley LC (2015). Vitamin C selectively kills KRAS and BRAF mutant colorectal cancer cells by targeting GAPDH. Science.

[18] Zhu XX, Ding YH, Y Wu, Qian LY, Zou H, He Q (2016). Silibinin: a potential old drug for cancer therapy. Expert Rev Clin Pharmacol.

[19] Sada N, Lee S, Katsu T, Otsuki T, Inoue T (2015). Targeting LDH enzymes with a stiripentol analog to treat epilepsy. Science.

[20] Khacha-Ananda S, Tragoolpua K, Chantawannakul P, Tragoolpua Y (2016). Propolis extracts from the northern region of Thailand suppress cancer cell growth through induction of apoptosis pathways. Invest New Drugs.

[21] Kakehashi A, Ishii N, Fujioka M, Doi K, M Gi, Wanibuchi H (2016). Ethanol-Extracted Brazilian Propolis Exerts Protective Effects on Tumorigenesis in Wistar Hannover Rats. PLoS One.

[22] Khoram NM, Bigdeli B, Nikoofar A, Goliaei B (2016). Caffeic Acid Phenethyl Ester Increases Radiosensitivity of Estrogen Receptor-Positive and -Negative Breast Cancer Cells by Prolonging Radiation-Induced DNA Damage. J Breast Cancer.

[23] Cao Z, Livas T, Kyprianou N (2016). Anoikis and EMT: Lethal “Liaisons” during Cancer Progression. Crit Rev Oncog.

[24] Bartolomé F, Abramov AY Measurement of mitochondrial NADH and FAD autofluorescence in live cells. Methods Mol Biol.

[25] Blacker TS, Duchen MR (2016). Investigating mitochondrial redox state using NADH and NADPH autofluorescence. Free Radic Biol Med.

[26] Stringari C, Edwards RA, Pate KT, Waterman ML, Donovan PJ, Gratton E (2012). Metabolic trajectory of cellular differentiation in small intestine by Phasor Fluorescence Lifetime Microscopy of NADH. Sci Rep.

[27] Cuyàs E, Corominas-Faja B, Menendez JA (2014). The nutritional phenome of EMT-induced cancer stem-like cells. Oncotarget.

[28] Miranda-Lorenzo I, Dorado J, Lonardo E, Alcala S, Serrano AG, Clausell-Tormos J, Cioffi M, Megias D, Zagorac S, Balic A, Hidalgo M, Erkan M, Kleeff J, Scarpa A, Sainz B, Heeschen C (2014). Intracellular autofluorescence: a biomarker for epithelial cancer stem cells. Nat Methods.

[29] Croce AC, Bottiroli G (2015). New light in flavin autofluorescence. Eur J Histochem.

[30] Cameron E, Pauling L (1976). Supplemental ascorbate in the supportive treatment of cancer: Prolongation of survival times in terminal human cancer. Proc Natl Acad Sci U S A.

[31] Nechuta S, Lu W, Chen Z, Zheng Y, Gu K, Cai H, Zheng W, Shu XO (2011). Vitamin supplement use during breast cancer treatment and survival: a prospective cohort study. Cancer Epidemiol Biomarkers Prev.

[32] Harris HR, Orsini N, Wolk A (2014). Vitamin C and survival among women with breast cancer: a meta-analysis. Eur J Cancer.

[33] Gao P1, Zhang H, Dinavahi R, Li F, Xiang Y, Raman V, Bhujwalla ZM, Felsher DW, Cheng L, Pevsner J, Lee LA, Semenza GL, Dang CV (2007). HIF-dependent antitumorigenic effect of antioxidants in vivo. Cancer Cell.

[34] Shaw FL, Harrison H, Spence K, Ablett MP, Simões BM, Farnie G, Clarke RB (2012). A detailed mammosphere assay protocol for the quantification of breast stem cell activity. J Mammary Gland Biol Neoplasia.

